# Effect of Storage Time of a Ceramic Primer on Microshear Bond Strength to Zirconia

**Published:** 2018-11

**Authors:** Amir Ghasemi, Alireza Sadr, Anahita Pourhashemi

**Affiliations:** 1 Professor, Department of Restorative Dentistry, School of Dentistry, Shahid Beheshti University of Medical Sciences, Tehran, Iran; 2 Associate Professor, Department of Restorative Dentistry, School of Dentistry, University of Washington, Washington, USA; 3 Assistant Professor, Department of Restorative Dentistry, School of Dentistry, Hormozgan University of Medical Sciences, Hormozgan, Iran

**Keywords:** Shear Strength, Ceramic Primer, Zirconium Oxide

## Abstract

**Objective:**

This study assessed the effect of shelf life of a ceramic primer containing 10-methacryloyloxydecyl dihydrogen phosphate (MDP) monomer on microshear bond strength of zirconia ceramic to composite resin.

**Materials and Methods:**

Sixty-four sintered zirconia samples (1.5×5×7 mm) were pretreated with Clearfil Ceramic Primer (CCP) at baseline and after one, two and six months of storage at 6°C. Composite cylinders were fabricated using Tygon tubes (1 mm height, 0.7 mm diameter) and placed on treated zirconia blocks, light-cured and subjected to microshear bond strength test. Mode of failure was determined under a stereomicroscope. Fourier-transform infrared spectroscopy (FTIR) was performed at each storage time point. Data were analyzed using one-way ANOVA and Tukey’s test.

**Results:**

Significant differences were noted in microshear bond strength after six months of storage compared to baseline (P<0.05). Significant differences were noted in FTIR spectra at the four time points (P<0.05).

**Conclusions:**

The effectiveness of one-component ceramic primer in bonding to zirconia was significantly affected by the storage of this material in a time-dependent manner. Visible new peaks and changes in FTIR spectra over time indicated alterations in the composition of ceramic primer affecting its shelf life.

## INTRODUCTION

Since 2010, a positive test for the anti-cyclic Dental application of zirconia-based ceramics has greatly increased due to favorable esthetics, optimal mechanical properties and biocompatibility [1]. Yttrium tetragonal zirconia polycrystal-based materials are now considered the core material of choice for all-ceramic restorations [2]. However, a strong and durable bond to zirconia is difficult to achieve [3]. Resins containing phosphoric acid ester monomer, 10-methacryloyloxydecyl dihydrogen phosphate (10-MDP) are currently widely accepted to enhance the bond to zirconia ceramics by increasing wettability and formation of strong hydrogen and covalent bonds [4]. This monomer has been known as one of the most effective functional monomers to achieve bonding not only to the tooth structure, but also to restorative materials and alloys [4].

On the other hand, bonding of resins to silicate-based ceramics has been enhanced by silane coupling agents. The adhesion of silane coupling agents and non-silica-based restorative materials such as alumina, zirconia or metals is weaker than the silica-based ceramics. The manufacturers have tended to introduce single-component primers that simplify pretreatment of the restorative materials for practitioners. Recently, multipurpose primers containing acidic monomers and silane in a single bottle were introduced. Clearfil Ceramic Primer (CCP; Kuraray Noritake Dental, Tokyo, Japan) is a single-component, silane and 10-MDP-containing primer commonly used to enhance the bond strength of zirconia ceramic to resin-based materials. The conventional single-bottle silane bonding agents, which contained silane coupling agent and a weak acid, had a major drawback of limited shelf life. Rapid evaporation and hydrolysis of the solvent [5] and formation of oligomers [6] have been reported as unfavorable changes in the composition of silane agents over time that adversely affect the bond strength of ceramics and compromise the success of silica-based restorations. However, the number of studies on the effect of shelf life of the newly introduced product on bond strength of zirconia ceramics is scarce.

The Fourier transform infrared spectroscopy (FTIR) is a technique commonly used to determine the molecular structure of materials and presence of functional groups. In other words, it reflects the organic and inorganic components in the composition of a material, qualitatively and quantitatively [7]. This study sought to assess the effect of shelf life of CCP on microshear bond strength of zirconia ceramic to composite resin. The FTIR was also performed to determine the changes in the composition of ceramic primer over time. The null hypothesis was that the bond strength and composition of CCP would not change over six months of storage in a refrigerator.

## MATERIALS AND METHODS

### Fabrication of zirconia ceramic blocks

Sixty-four zirconia blocks (Mamut Dental Inc., Hong Kong, China) measuring 1.5 mm × 5 mm × 7 mm were fabricated using a thin sectioning saw (Hamko, NY, USA). Samples were polished using 600- and 800-grit silicon carbide abrasive papers (3M ESPE, St. Paul, MN, USA) and sintered in a furnace at 1500°C (LHT; Nabertherm, Lilienthal, Germany). The blocks were randomly divided into four groups (n=16) for bond strength assessment.

### Microshear bond strength:

CCP was purchased fresh from the manufacturer. The composition and batch number of CCP are presented in Table 1.

**Table 1.** List of materials used in this study and their composition

** Material **	** Chemical composition **	** LOT no. **	** Manufacturer **
Clearfil Ceramic Primer (CCP)	Ethanol, 10-MDP, γ-MPTS	CJ0005	Kuraray Noritake Dental, Tokyo, Japan
Valux (composite resin)	Resin: Bis-GMA, TEGDMA silica or zirconia, Filler: 66vol% photo-initiator	N463727	3M ESPE, St. Paul, MN, USA

10-MDP: 10-Methacryloyloxydecyl dihydrogen phosphate, γ-MPTS: 3-trimethoxysilylpropyl methacrylate, Bis-GMA: Bisphenol A diglycidyl ether methacrylate, TEGDMA: Triethylene glycol dimethacrylate.

One CCP bottle was used immediately and then kept refrigerated at 6°C for the bond strength study period. Zirconia specimens in groups 1, 2, 3 and 4 were treated with CCP at baseline and after one, two and six months of CCP storage. The following procedures were similarly performed in the four groups:

CCP was applied to the surface of zirconia substrates by a microbrush for 60 seconds according to the manufacturer’s instructions and air-dried for 15 seconds. A hybrid composite (Z100 Valux Plus, 3M ESPE, St. Paul, MN, USA) was applied into silicon Tygon tubes (Tygon Norton Performance Plastic Co., Cleveland, OH, USA) on the surface of zirconia substrates with 1 mm height and 0.7 mm internal diameter. The composite was light-cured for 40 seconds using a light-curing unit (Demetron LC; Kerr, Orange, CA, USA). After one hour, the Tygon tubes were removed using a stainless steel feather blade, such that a composite cylinder (1 mm × 0.7 mm) remained bonded to the zirconia surface. The specimens were incubated at 37°C and 100% humidity for 24 hours and were then subjected to microshear load in a desktop testing machine (Bisco, Schaumburg, IL, USA). Each zirconia block was attached to the jig by cyanoacrylate glue. A stainless steel wire (0.2 mm in diameter) was used to connect the load cell to the composite cylinder, and load was applied at a crosshead speed of 0.5 mm/minute until fracture. The maximum load at fracture was recorded in Newtons (N) and was then converted to megapascals (MPa).

The fracture surfaces were then evaluated under a stereomicroscope (SZX16; Olympus, Tokyo, Japan) at ×40 magnification to determine the mode of failure. Mode of failure was categorized into three groups of adhesive (at the zirconia-composite interface), cohesive (within the composite resin) and mixed (a combination of adhesive and cohesive failures).

The Kolmogorov-Smirnov test and the Shapiro-Wilk test were used to assess the normal distribution of data. The microshear bond strength data were analyzed using one-way ANOVA. Post-hoc Tukey’s test was applied for pairwise comparisons of the groups, at a significance level of 0.05.

### The FTIR analysis:

For FTIR analysis, CCP was applied to NaCl plates and air-dried to form a thin layer. A ceramic primer bottle with a production date similar to that of the bottle used for microshear bond strength testing was used for analysis. The plates were allowed three minutes after applying CCP in order for the solvent to evaporate, and were then subjected to FTIR (Tensor 27; Bruker, Karlsruhe, Germany). The FTIR spectra with a resolution of 4 cm^−1^ in the range of 400–4000 cm^−1^ were recorded. The FTIR analysis was repeated at the four time points (baseline and after one, two and six months of storage) to assess the effect of shelf line of the ceramic primer on its composition.

## RESULTS

### Results of microshear bond strength test:

The mean (± standard deviation) bond strength values were 25.88±4.00, 25.43±7.08, 23.02±4.77 and 18.38±5.68 MPa at baseline and at one, two and six months, respectively.

The Kolmogorov-Smirnov test confirmed the normal distribution of data (P>0.05). Thus, one-way ANOVA was applied, which showed significant differences among the study groups in terms of bond strength (P=0.001). The post hoc Tukey’s test (Table 2) revealed that the difference in bond strength at baseline and after one and two months was not significant (P>0.05); however, significant differences were noted at six months compared to the baseline and one-month storage groups (P<0.05).

**Table 2.** Pairwise comparisons of groups using post-hoc Tukey’s test (P value)

** Study groups **				
	** Baseline **	** One month **	** Two months **	** Six months **
** Study groups **				
** Base line **	-	0.99	0.46	0.002 ^*^
** One month **	0.99	-	0.6	0.003 ^*^
** Two months **	0.46	0.6	-	0.9
** Six months **	0.002 ^*^	0.003 ^*^	0.09	-

* P≤0.05 was considered statistically significant.

Table 3 shows the frequency of modes of failure; mixed failure had the highest frequency.

**Table 3.** Modes of failure at the four time points

** Mode of Failures **			
	** Mixed N(%) **	** Adhesive N(%) **	** Cohesive N(%) **
** Study Group **			
** Baseline **	11 (68.75)	1 (6.25)	4 (25
** One month **	10 (62.5 )	4 (25)	2 (12.5)
** Two months **	11 (68.75)	4 (25)	1 (6.25)
** Six months **	9 (56.25)	7 (43.75)	0

**Fig. 1.**
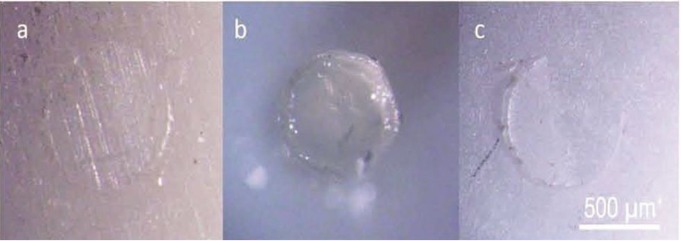
Representative light-microscopic images of the failure modes; a) adhesive failure; b) Cohesive failure; c) mixed failure

Representative images of each mode of failure are presented in Figure 1(a–c).

### Results of FTIR analysis:

**Fig. 2.**
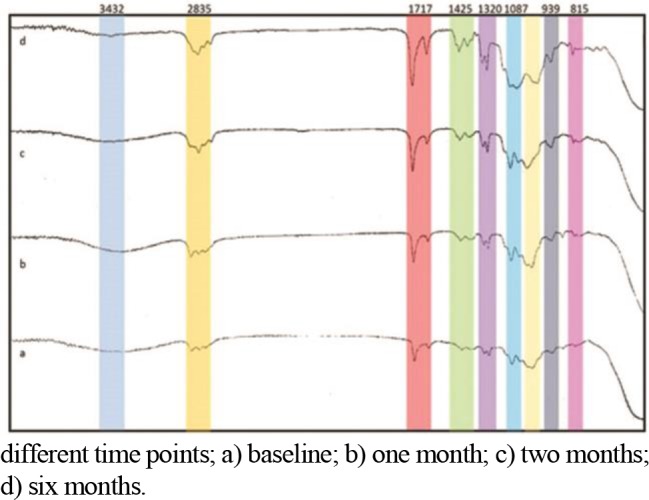
FTIR spectra of the ceramic primer solution tested at different time points; a) baseline; b) one month; c) two months; d) six months.

Figure 2 shows changes in FTIR spectra and the peaks representing the bonds over time. A new peak at 3432 cm^−1^ developed during the six-month study period, which was attributed to the formation of OH bond in Si-OH. The intensity of the peak at 3432 cm^−1^ gradually increased during the six-month period.

A reduction in the intensity of the peak at 815 cm^−1^ was noted over time, which was due to the disintegration of Si-O-CH_3_ bond in silane. Also, the peak at 2835 cm^−1^ gradually disappeared over time, and at six months no peak was observed at 2835 cm^−1^ (due to the disintegration of Si-O-CH_3_ bond). This peak might have also belonged to C-H bond in ethanol, which disappeared over time (due to solvent evaporation). The peak at 902 cm^−1^ was attributed to the formation of Si-OH bond, which became more prominent over time.

The peak at 1087 cm^−1^ belonged to the formation of Si-O-Si bond, which became more distinct over time. This indicates disintegration of activated silane and bond of silanol functional group to the silanol group of the coupling agent. The peak at 1717 cm^−1^ was attributed to the presence of 10-MDP group, which gradually deceased over time.

## DISCUSSION

Silane coupling agents are extensively used in dentistry. These primers contain acid, water and alcohol or another solvent. The role of acid is to hydrolyze the functional groups of silane to silanol, and the role of solvent is to preserve the solubility of the coupling agent.

In hydrolyzed silane, monomeric or oligomeric silanols may react with other coupling agents or superficial silanols to form siloxane bonds or remain as free silanols. Adding any other compound like acidic monomers to ceramic primers may complicate the mixture [8].

This study aimed to assess the effect of shelf life of a MDP-containing primer on the bond strength of ceramic to composite. In the current study, microshear bond strength was measured, which is superior to microtensile test since the former is reproducible. Moreover, shear forces are numerous in the oral cavity. Also, microshear test does not cause preload failure since there is no need for cutting of specimens. The results showed that the mean bond strength was 25.88 MPa at baseline, which is almost similar to the values reported at baseline by some other studies [9, 10].

Conversely, lower and higher values were reported by Keul et al, [11] in 2013, Matinlinna et al, [12] in 2007 and Lehmann et al, [13] in 2009. This controversy is probably due to the fact that they prepared the primer themselves and tested the samples 24 hours after the application of primer; whereas, we used the commercially available ceramic primer, which has certainly undergone many chemical reactions since its production date. Also, higher bond strength values may be attributed to surface treatment of samples, which was not done in our study. Lower bond strength values reported in previous studies [11, 12] may be attributed to the use of different testing machines, operator’s performance, and the type of test and substrate. Moreover, absence of 10-MDP in the formulation of some primers results in lower bond strength. In our study, the mean bond strength was 25.43 MPa at one and 23.02 MPa at two months, which were not significantly different from the bond strength value at baseline. This finding was in line with the results of Hooshmand et al, [14] in 2004. Ikemura et al. [10] found no significant difference in bond strength at two months and at baseline. This means that major changes did not occur in a short period of time and bond strength was not affected. However, Matinlinna et al, [15] in 2006 and Keul et al, [11] in 2013 reported significant reductions in bond strength values at one and two months compared to baseline. Sekitani et al, [16] in 2009 also showed such significant reductions in bond strength after two weeks. On the other hand, because of using different types of silane with different pH values, hydrolysis patterns, condensation processes, and the effectiveness of silane might have been different.

At six months, bond strength was 18.38 MPa in our study, which was significantly different from the values at baseline and at one month. However, bond strength at six months was not significantly different from the value at two months. Similar to our study results, Lehmann et al, [13] in 2009 reported a significant reduction in bond strength at five months compared to baseline.

Prominently higher frequency of cohesive failure and fewer adhesive failures at baseline in contrast to no cohesive failure and highest frequency of adhesive failure at six months confirmed our stereomicroscopic findings.

Regarding the results of FTIR in our study, a new peak formed at 3432 cm^−1^ during the six-month study period, which belonged to the formation of the O-H bond in Si-OH and water. The intensity of the peak at 3432 cm^−1^ increased during the six months but a reduction in the peak at 815 cm^−1^ was noted, which was due to the disintegration of Si-O-CH_3_ in silane. Also, the peak at 2835 cm^−1^ gradually disappeared during the six-month period, which can be due to the disintegration of Si-O-CH_3_ or the C-H bond in ethanol, indicating solvent evaporation. The peak at 902 cm^−1^ was attributed to the formation of the Si-OH bond, which became more prominent over time (as expected). Also, the peak at 1087 cm^−1^ was attributed to the formation of the Si-O-Si bond and became more distinct over time. These findings are in agreement with those of Matinlinna et al, [12] in 2007, Aksornmuang et al, [17] in 2004 and Ishida [18] in 1987. The intensity of the peak at 1717 cm^−1^ decreased over time; this peak was attributed to the carbonyl methacrylate group of 10-MDP; this result was in line with the findings of Feitosa et al, [19] in 2014 who evaluated the chemical interactions of 10-MDP. In our study, the primary formulation of silane changed over time and it was converted to silanol. In fact, formation of the Si-O-Si bond and increased intensity of its peak at 1087 cm^−1^ indicate disintegration of activated silane. The formed silanol group bonds to the silanol in the composition of the coupling agent, indicating disintegration of the primer. Following this, the CH_3_-O bond would no longer be present in the formulation of primer and thus, its related peak disappears on the FTIR analysis. Instead, an O-H bond forms. Our study showed that during the six-month period, significant changes occurred in the primer. Many molecular bonds were broken and their related peaks were disappeared. However, it should be noted that we used solvents, which were thought to have significant effects on bonds over time. The acid present in the primer causes hydrolysis of the silane ester (Si-O-CH_3_) and its conversion to Si-OH, which indicates conversion of silane to silanol. It seems that over time, Si-O-Si bonds form and produce high molecular weight oligomers. The results of FTIR showed hydrolysis and subsequent destruction of solution and confirmed the findings of microshear bond strength test. These findings are in agreement with those of several previous studies [9, 20]. They reported that disintegration and hydrolysis of single-component silanes occur more frequently than two-component primers; the negative impact of this phenomenon would be the formation of oligomers that can decrease the efficacy of silane over time. Considering the limited number of studies on the effect of time on single-bottle primers, future studies with longer durations are required to better elucidate this topic.

## CONCLUSION

Within the limitations of this study, the results showed that passage of time significantly affected the bond strength of zirconia to composite mediated by ceramic primer. Over time, the primer was hydrolyzed and the bonds in the formulation of silane and 10-MDP were broken and replaced with bonds involving silanol and siloxane molecules. Disintegration of the primer and 10-MDP monomer (shown by FTIR) confirmed the reduction in microshear bond strength observed during the six-month study period.
